# Concentration Regimes of Biopolymers Xanthan, Tara, and Clairana, Comparing Dynamic Light Scattering and Distribution of Relaxation Time

**DOI:** 10.1371/journal.pone.0062713

**Published:** 2013-05-06

**Authors:** Patrícia D. Oliveira, Ricardo C. Michel, Alan J. A. McBride, Angelita S. Moreira, Rosana F. T. Lomba, Claire T. Vendruscolo

**Affiliations:** 1 Biotechnology, Technology Development Centre, Federal University of Pelotas, Pelotas, Rio Grande do Sul, Brazil; 2 Eloísa Mano Macromolecules Institute, Federal University of Rio de Janeiro, Rio de Janeiro, Rio de Janeiro, Brazil; 3 Chemical, Pharmaceutical, and Food Science Centre, Federal University of Pelotas, Pelotas, Rio Grande do Sul, Brazil; 4 Well Engineering Technology Research and Development Centre, Leopoldo Américo Miguez Mello, Petrobras, Rio de Janeiro, Brazil; Max Planck Institute for Polymer Research, Germany

## Abstract

The aim of this work was to evaluate the utilization of analysis of the distribution of relaxation time (DRT) using a dynamic light back-scattering technique as alternative method for the determination of the concentration regimes in aqueous solutions of biopolymers (xanthan, clairana and tara gums) by an analysis of the overlap (c*) and aggregation (c**) concentrations. The diffusion coefficients were obtained over a range of concentrations for each biopolymer using two methods. The first method analysed the behaviour of the diffusion coefficient as a function of the concentration of the gum solution. This method is based on the analysis of the diffusion coefficient versus the concentration curve. Using the slope of the curves, it was possible to determine the c* and c** for xanthan and tara gum. However, it was not possible to determine the concentration regimes for clairana using this method. The second method was based on an analysis of the DRTs, which showed different numbers of relaxation modes. It was observed that the concentrations at which the number of modes changed corresponded to the c* and c**. Thus, the DRT technique provided an alternative method for the determination of the critical concentrations of biopolymers.

## Introduction

An important aspect in polymer materials research is the relationship between the concentration and the static/dynamic properties of the macromolecule, in particular, the behavioural changes in solutions ranging from the dilute, semi-dilute to the concentrated regime [1], [2]. The determination of the possible concentration regimes allows for an understanding of the biopolymer behaviour in solution and thus potential applications. There are three dynamic concentration regimes for a polymer solution in a good solvent: diluted, semi-diluted and concentrated [3]. The difference between the regimes is based on the interactions of the biopolymer in solution. Polysaccharides in solution tend to adopt a random coil conformation that fluctuates continually due to Brownian motion. At low concentrations the individual chains of a biopolymer assume a random coil conformation, separate from each other and that move independently. As the concentration increases, the molecules begin to interact and may become more stable by overlapping and binding with other molecules in the solution. Under these conditions the individual chains have difficulty moving due to interactions with neighbouring chains [4]. The concentration at which the individual chains begin to physically interact is known as the overlap concentration (c*) [3]. The concept of c* is based on the theory that polymer coils in solution are in a stationary state, but they occupy a hydrodynamic volume that, when above the critical concentration, packs the molecules together [1]. Southwick *et al.* defined a solution as being dilute when the intermolecular overlap does not interfere with the translational diffusion of molecules, in other words, when c<c* [5].

The concentration increase associated with a semi-dilute regime results in a contraction of the molecules in solution. The transition from a semi-dilute to a concentrated regime occurs at the critical concentration of aggregation (c**), whereby the molecules can no longer contract and further increases in concentration result in the formation of aggregates [6]. The solution is semi-dilute when the individual chains (radius of gyration) overlap and become entangled, greatly reducing their mobility. The most common techniques used for the determination of c* and c** are rheological analysis and light scattering. Dynamic light scattering (DLS) can be used to trace the diffusivity of molecules in solution over a range of concentrations [1], [7]–[9]. Native and modified xanthan samples have been studied using both static and DLS and there are reports of ambiguity in the determination of c* for semi-flexible molecules [8]. Suggesting that the c* is dependent on the experimental technique used and the interpretation of the data.

Xanthan gum is an extracellular polysaccharide produced by the fermentation of bacteria belonging to the *Xanthomonas* genera. Commercial xanthan gum is usually extracted from cultures of *X. campestris* pathovar (pv) campestris, however, other species can produce xanthan gum, including *X. phaseoli, X. juglandis* and *X. arboricola*. The xanthan used in the present study was produced by *X. arboricola* pv pruni strain 06 [10], [11]. *Beijerinckia* spp. can produce exopolysaccharides with specific compositions that vary according to the species [12]. The biopolymer synthesized by *Beijerinckia* spp. strain 7070 is called clairana. The chemical composition of clairana has been determined [13] and its production and properties have been extensively investigated [14]–[19]. Tara gum is a less thoroughly studied galactomannan. It is obtained from the endosperm of the seeds of the tara tree (*Caesalpinia spinosa*) [20]. Tara gum is a neutral polysaccharide and aqueous solutions are disordered with some degree of rigidity [21]. They are usually described as semi-flexible polymers with a random coil conformation that, under controlled conditions, can form interacting solutions [22].

The main objective of this work was to evaluate the utilization of analysis of the distribution of relaxation time (DRT) using a dynamic light back-scattering technique as alternative method for the determination of the concentration regimes in aqueous solutions of biopolymers (xanthan, clairana and tara gums) by an analysis of the overlap (c*) and aggregation (c**) concentrations.

## Materials and Methods

### Culture Conditions and Biopolymer Production

The xanthan gum was produced using *X. arboricola* pv pruni strain 06 (EMBRAPA, Pelotas, Brazil). *X. arboricola* pv pruni was maintained in YM medium [23] and was stored at 4°C [24]. The clairana gum was produced by *Beijerinckia* spp. strain 7070, which was originally isolated from sugar cane soil [25]. Briefly, the seed cultures were grown in YM medium, incubated in an orbital shaker (New Brunswick Scientific, model Innova 4230) at 28°C. Biopolymer production was performed in a 10 L bioreactor (B. Braun Biotech. Inc., Biostat B model) containing 7 L of production medium. For xanthan production the medium described in patent WO/2006/047845 [26] was used and the medium for clairana production was as described in patent n° PI 0105856-8 [25]. The following operational conditions were used: 400 rpm stirrer speed, 1 vvm air flow rate, 28°C and the fermentation time was 72 h. The post-fermentation processing included centrifugation at 16,000 g for 45 minutes (RC-5C, Sorval Instruments). The biopolymers were recovered by adding ethanol (96%) to the supernatant to precipitate the polymers. The resulting polymeric fibres were recovered and dried at 56°C until constant weight and triturated in a disk mill (Fritsch, model Pulverisette) to a particle size of 60–150 mesh. The tara gum was supplied by Metachem Industrial e Commercial Ltda.

### Preparation of the Biopolymer Solutions

The three biopolymer samples were prepared by solubilization in deionized water (stock concentration 5 g.L^−1^) and stirred for 16 h at 50°C. Sodium azide (1×10^−3^ g.L^−1^) was added to prevent microbial contamination of the solutions. To remove dust particles and aggregates, the samples were filtered through membranes with pore sizes ranging from 3.0 to 0.45 µm, (Millipore, Merck Millipore). Starting from the stock solution, dilutions were prepared, using the same solvent, with stirring. The concentrations of the solutions were varied within a range that included the different dynamic regimes of concentration, and these were checked gravimetrically.

### Determination of Dynamic Light Scattering (DLS)

The light scattering measurements were carried out using a Malvern Nanosizer ZS. Samples were analysed in a glass cuvette with a minimum of 3 repetitions. All analyses were performed at 25°C. The angle of detection of the scattered light was 173°, as determined by back-scatter. The Nanosizer ZS used a 4 mW He-Ne laser, with an operating wavelength (λ_0_) of 633 nm. Changes in the solvent and the sample viscosities, refractive index and absorption were not evaluated and were considered to be 0.8872 mPa·s, 1.330 and 0.001, respectively. The values of the coefficients of translational diffusion were obtained with the multimodal algorithm CONTIN, provided in the software package Dispersion Technology Software 5.0 (Malvern Instruments Ltd., Worcestershire, U.K.).

## Results and Discussion

### Evaluation of the Average Coefficients of Translational Diffusion

The literature contains several different methodologies for the determination of critical concentration, which may be either observational or theoretical. Tinland *et al*. [9] estimated c* and c** using equations. Southwick *et al*. [7] determined c* using two methodologies, the observed values were based on the slope of the diffusion coefficient *versus* the solution concentration curve. Rodd *et al*. [1] determined the c* by estimating a 5% variation in the diffusion coefficient compared to the value obtained in infinity dilution, the variation from the plateau of diffusion. In the present work, the methodology used was observational. The critical concentrations were determined at the points where changes occurred in the slope of the diffusion coefficient *versus* the concentration curve of the biopolymer. The c* value was determined at the beginning of the plateau and the c** value was determined by finding the maximum diffusion coefficient. Note, the diffusion coefficient is a physical quantity and in the DLS technique, motion is not dependant on the scattering angle used to measure the response. In the DLS experiments carried out by Rodd and colleagues the angle used was 40°, and the diffusion coefficient *versus* the concentration curve showed a sharp drop in the diffusion coefficient [1]. However, at angles between 60° and 100°, this drop was not observed. In the present study, similar behaviour was observed at an angle of 173°, in agreement with the previously data [1].

**Figure 1:**
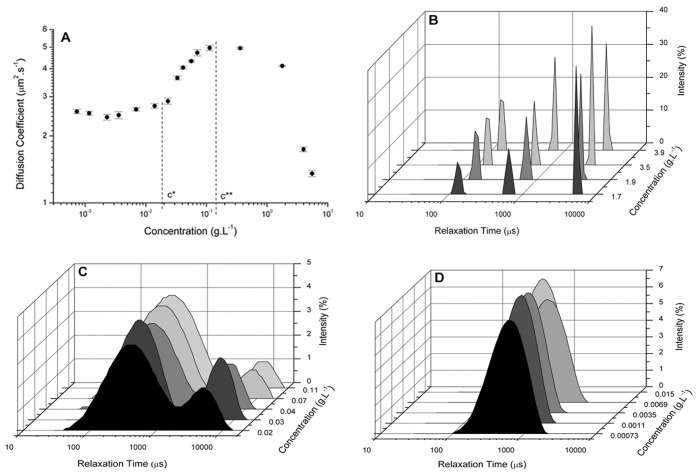
Plot of the diffusion coefficient versus biopolymer concentration and the light scattering intensity as a function of the distribution in the relaxation times (DRT) for tara gum. (A) The mean diffusion coefficient (± SEM) of a range of aqueous biopolymer solutions (5–5.0×10^−4^ g.L^−1^) are presented. The c* value (based on the plateau) and the c** value (equivalent to the maximum diffusion coefficient) are indicated by the dotted lines. The graphs represent solutions at different concentration regimes: (B) concentrated, showing a trimodal distribution, while the (C) semi-diluted and the (D) diluted shown a bimodal and a monomodal distribution, respectively.

At very dilute concentrations (c → 0), a plateau in the diffusion coefficient was observed at approximately 2.5 µm^2^·s^−1^, Fig. 1A. Increasing the concentration caused a rise in the diffusion coefficient up to a certain concentration (1.6×10^−1^ g.L^−1^). From this point onwards, increasing the concentration resulted in a lowering of the diffusion coefficient. Thus, it was possible to determine the concentration regimes and, consequently, the c* and c**.

The critical concentrations of tara gum were 1.8×10^−2 ^g.L^−1^ for the c* and 1.3×10^−1 ^g.L^−1^ for the c**. Thus, the concentration range for the dilute solution was c <1.8×10^−2 ^g.L^−1^, semi-dilute was 1.8×10^−2 ^g.L^−1^< c <1.3×10^−1 ^g.L^−1^ and concentrated was c >1.3×10^−1 ^g.L^−1^. The production of tara gum is restricted to Peru, its country of origin. For this reason, the marketing and consequently the study of this gum remain relevant. The critical concentrations c* and c** as determined by light scattering have not been reported previously. However, rheological analysis has been used to determine the critical concentrations. Previously, the c** for tara gum was reported to be 5.2 g.L^−1^ and that for locust gum was 7.0 g.L^−1^ in one report [27] while another reported a value of 2.4 g.L^−1^ [28]. The c* for guar gum was shown to be 0.55 g.L^−1^ and the c** was 2.8 g.L^−1^ [29], while another study reported 4.0 g.L^−1^ for c** [30].

**Figure 2:**
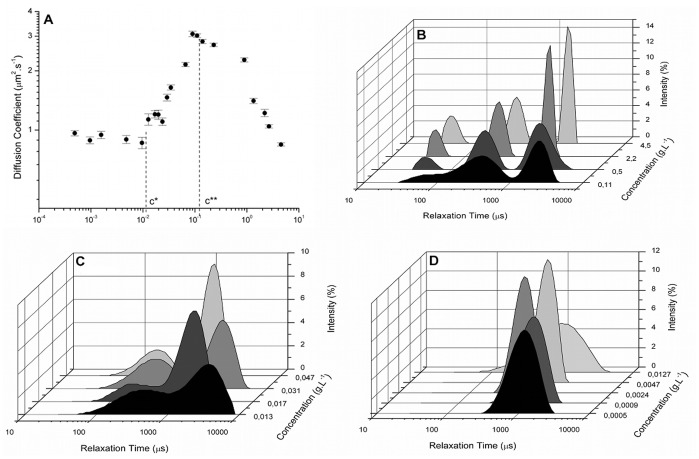
Plot of the diffusion coefficient versus biopolymer concentration and the light scattering intensity as a function of the distribution in the relaxation times (DRT) for xanthan. (A) The mean diffusion coefficient (± SEM) of a range of aqueous biopolymer solutions (5–5.0×10^−4^ g.L^−1^) are presented. The c* value (based on the plateau) and the c** value (equivalent to the maximum diffusion coefficient) are indicated by the dotted lines. The graphs represent solutions at different concentration regimes: (B) concentrated, showing a trimodal distribution, while the (C) semi-diluted and the (D) diluted shown a bimodal and a monomodal distribution, respectively.

The behaviour and properties of xanthan solutions at different concentration regimes have been studied extensively [7], [29], [31]–[34]. The diffusion coefficient *versus* the concentration curve showed a plateau in the diffusion coefficient as the concentrations approached zero, at 0.9 µm^2^.s^−1^, Fig. 2A, similar to that reported previously [1]. At increasing concentrations, the diffusion coefficient increased until a certain concentration, at which it decreased. The critical concentrations determined for xanthan were 1.3×10^−2 ^g.L^−1^ for the c* and 1.1×10^−1 ^g.L^−1^ for the c**. Thus, the concentration range for the dilute solution is c <1.3×10^−2 ^g.L^−1^, for semi-dilute it is 1.3×10^−2 ^g.L^−1^< c <1.1×10^−1 ^g.L^−1^ and the solution was concentrated when c >1.1×10^−1 ^g.L^−1^. A summary of the xanthan critical concentrations available in the literature is presented, Table 1. The c* and c** values reported in this study were lower than those previously published using rheological analysis and/or DLS. A potential limitation of the current study is that changes in the viscosity of the biopolymer solutions and their impact on the diffusion coefficient were not evaluated. However, a previous report that evaluated the impact of increasing viscosity reported a similar change in the diffusion coefficient (∼2 fold increase) over a 5-fold change in concentration [1].

**Table 1:** Values for the critical concentrations (c* and c**) for xanthan solutions, reported in the literature.

Analysis	c* (g.L^−1^)	c** (g.L^−1^)	Authors
DLS	1.15×10^−1^ (theoretical) 1.25×10^−1^ (observed)	7.0×10^−1^	Southwick *et al*. [7]
DLS	4.0×10^−1^	–	Tinland *et al*. [9]
DLS	5.0×10^−2^	7.0×10^−1^	Rodd *et a*l. [1]
DLS	6.0×10^−2^	–	Rodd *et al.* [37]
DLS	–	6.0×10^−1^	Nash *et al*. [6]
Rheology	3.0×10^−1^	1.1	Cuvelier & Launay [32]
Rheology	1.26 (native)	6.0 (native)	Milas *et al*. [33]
	1.0 (renatured)	7.8 (renatured)	
Rheology	–	2.0	Meyer *et al*. [42]
Rheology	6.25×10^−1^	–	Esquenet & Buhler [34]

The differences in the c * and the c ** values are related to the chemical structures of the biopolymers, and this can affect their molar mass and hydrodynamic radius. This affects their movement in a given solvent, and hence the calculation of the c * and the c**. The difference in the c* and c** values obtained by light scattering compared to those determined by rheological analysis may be due to the shear that these analyses required. For xanthan solutions, the shear forces dominated the Brownian motion at low shear rates [35]. This causes molecular alignment, resulting in a solution that does not exhibit true random movement. Doi and Edwards [3] demonstrated that, theoretically, under the application of shear forces, rod-like molecules could align in solution before molecular interaction. This alignment of the molecules caused a reduction in the volume fraction that was in the direction of shear and consequently, c* and c** occurred at higher concentrations. Although xanthan molecules are not rod-like in shape [36], their extended form in solution suggested that molecule alignment may occur. Thus, the semi-flexible nature of xanthan makes the determination of c* difficult when shear is applied [1]. The xanthan overlap concentrations were lower than those previously reported, as determined by DLS [7], [9]. This difference was likely due to the different methodologies used to determine the c*. Of note, the c* value was the same order of magnitude as those values previously [1], [37]. Furthermore, the c** value determined was the same order of magnitude as those determined by DLS [1], [6], [7].

**Figure 3:**
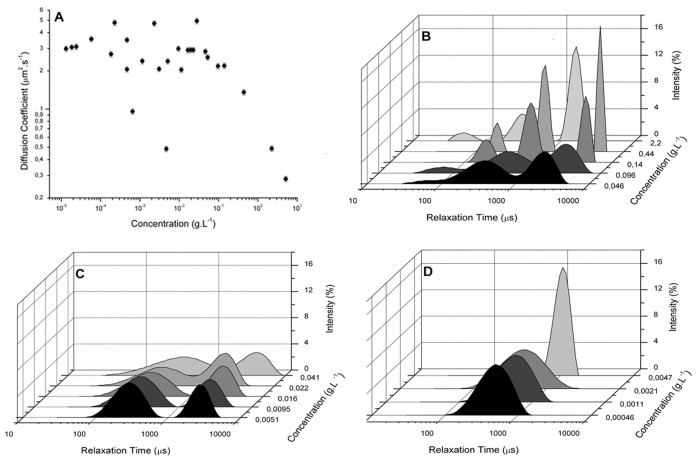
Plot of the diffusion coefficient versus biopolymer concentration and the light scattering intensity as a function of the distribution in the relaxation times (DRT) for clairana gum. (A) The mean diffusion coefficient (± SEM) of a range of aqueous biopolymer solutions (5–5.0×10^−4^ g.L^−1^) are presented. The c* value (based on the plateau) and the c** value (equivalent to the maximum diffusion coefficient) are indicated by the dotted lines. The graphs represent solutions at different concentration regimes: (B) concentrated, showing a trimodal distribution, while the (C) semi-diluted and the (D) diluted shown a bimodal and a monomodal distribution, respectively.

The diffusion coefficient versus concentration curve for clairana contained several large variations in the diffusion coefficient values (Fig. 3A). Such, that it was not possible to determine the c* or the c** for clairana.

In our work, it was observed that at very diluted concentrations (c → 0), the translational diffusion coefficient did not change with increasing concentration (Fig. 1A and Fig. 2A). The molecules could move freely because interactions did not occur between the molecules. Very dilute macromolecule solutions in a good solvent have a tendency to exclude all others based on the volume that they occupy. This is known as the excluded volume, and it is defined as the result of repulsion between polymer molecules due to space requirements. In this case, the average physical properties of the solution were not changed, therefore the translational diffusion coefficient remained the same [3].

The semi-diluted solution was in the concentration range: c*<c<c**. In this system, the effect of excluded volume disappeared because of the high concentration of polymer chains and Brownian motion was responsible for the hydrodynamic interactions. This motion caused friction at other regions of the polymer chain, mediated by the solvent used. Interaction between the fluidic parts of the polymer chain is a very dynamic process that includes polymer diffusion. By assuming that these interactions do not occur, it is possible to analyse the polymer chain as a whole and then apply the blob model. In this model, the polymer chain is viewed as a set of spheres or blobs, where the movement of each blob does not correlate with the movement of the others [38].

The dynamics of a polymer chain surrounded by other chains in a semi-dilute concentration regime can be explained by the reptation model if the molecules are in a disordered conformation [39]. Considering that the xanthan and tara gums, when in aqueous solution and under certain conditions, are semi-flexible polymers in a disordered conformation [4], [21], [22], their dynamics can be explained by reptation. The conformation and degree of flexibility of clairana was not studied. In reptation, it is assumed that a specific chain suffers reptation due to entanglement with the surrounding chains. Above the c*, diffusion reptation becomes the dominant mechanism in polymer solutions. As the concentration increases, different chains interact more frequently, causing a rise in the coefficient of diffusion of each blob. According to de Gennes [40], the main acting phenomenon is a decrease in blob size with increasing concentration because the higher number of molecules in solution promotes the occurrence of intermolecular interactions, as well as the formation of interlaced structures and structural contraction. As. It is thought that the c** depends on the type of polymer, especially its inherent stiffness, its molar mass and the quality of the solvent [29]. Milas *et al*. speculated that the c** was the beginning of a concentration range in which a uniform distribution of polymer segments was present in the solution volume [33].

The transition point of a semi-dilute solution is known as the c**, at which point the molecules have reached their maximum contraction, resulting in the formation of aggregates [6]. In concentrations above the c**, polymer-polymer interactions occur, which reduces the mobility of the molecules in solution. Thus, with a gradual increase in concentration, a decrease in the translational diffusion coefficient occurs, and it has been proposed that this behaviour represents the concentrated regime.

### Evaluation of the Distribution of Relaxation

The relaxation or correlation time of a property represents the time of the characteristic decay of the property [41], and it is the same as 1/Dq^2^, where D is the diffusion coefficient, and q is the scattering vector. For the DLS technique, it is known that the process of translational diffusion of a particle depends on its size; large particles have low diffusion rates and small particles have high diffusion rates. In this way, the different sizes of the particles present in a sample will generate different diffusion processes. These different diffusion processes correspond to a certain percentage of particles present in the solution and they have different relaxation times, resulting in different numbers of modes in the relaxation time. Polyelectrolyte solutions of biopolymers such as xanthan solutions at a semi-dilute concentration, display two modes in the relaxation time, and are related to fast and slow diffusion modes [1], [6], [7], [34], [36]. However, there are no reports on relaxation time modes in concentrated regimes. Thus, the effect of variations in the concentrations of the biopolymer solutions was evaluated by finding the DRTs based on the intensity of scattered light.

Figure 1(B, C, and D) shows the DRT as a function of the intensity of scattered light for several concentrations of tara gum. It was observed that each concentration regime corresponded to a different number of modes in the DRT. The concentrated regime (Fig. 1B) had a trimodal distribution, the semi-dilute regime (Fig. 1C) had a bimodal distribution and the diluted regime (Fig. 1D) exhibited a monomodal DRT. In each concentration regime, a reduction in solution concentration caused an enlargement in the modes present in the distribution curves and in the approximation among those modes. Using this approach, the modes progressed until one of the modes was suppressed. The concentration at which this suppression occurred corresponded to the critical concentration border. The transition from three to two modes of relaxation times corresponded to c**, while the transition from two to one mode corresponds to the c*.

The DRTs obtained for the xanthan sample (Fig. 2B, C and D), exhibited the same behaviour as that described for tara gum (Fig. 1). Southwick et al. [19] reported that low concentrations had a monomodal relaxation time, but at concentrations above the c**, the solutions exhibited bimodal behaviour. This was in agreement with the observations described by Nash *et al*., [6] and Esquenet and Buhler [34]. The presence of a third DRT mode at concentrations above 0.06% wt.% was observed by Nash and co-workers [6]. However, this was not related to changes in the concentration regime of the biopolymer solution. The authors believe that the current study is the first to observe this relationship.

It was observed that at the c* and c**, the DRT corresponded to the overlap and aggregation concentrations, as determined by the coefficient of translational diffusion versus concentration. This can be seen in Fig. 1A and 2A, for the tara gum and xanthan samples. This suggests that the evaluation of the DRT can be used as an alternative to the evaluation of the coefficient of translational diffusion for the determination of the c * and c** values. To test this proposal, the DRT method was used with clairana, for which the c* and c** could not be determined by an evaluation of the curve of the coefficient of translational diffusion versus concentration.

Figure 3 (B, C and D) presents the DRT versus concentration for clairana gum, separated by the number of relaxation modes. It was observed that a decrease in the number of relaxation modes occurred at the following concentrations: 4.1×10^−2 ^g.L^−1^, corresponding to the c** and 4.7×10^−3 ^g.L^−1^, corresponding to the c*. From these results, the intervals for the three concentration regimes of clairana were defined. Thus, it was determined that the solution was in a diluted regime at c <4.7×10^−3 ^g.L^−1^, semi-diluted at 4.7×10^−3 ^g.L^−1^< c <4.1×10^−2 ^g.L^−1^ and concentrated at c >4.1×10^−2 ^g.L^−1^. The DRT curve shows the different relaxation modes, which represent distinct values of diffusion coefficients.

In conclusion, only the DRT method was capable of determining the critical concentrations c* and the c**, as well as the concentration regimes for all of the polysaccharide polymers tested in this study. The c* and the c** for clairana gum could not be determined from the curve of the translational diffusion coefficient as a function of concentration as determined by DLS. These findings suggest that the DRT can be used as an alternative method for the determination of the c* and the c**.
